# Intensity Matters for Musculoskeletal Health: A Cross-Sectional Study on Movement Behaviors of Older Adults from High-Income Scottish and Low-Income South African Communities

**DOI:** 10.3390/ijerph18084310

**Published:** 2021-04-19

**Authors:** Ilaria Pina, Amy E. Mendham, Simone A. Tomaz, Julia H. Goedecke, Lisa K. Micklesfield, Naomi E. Brooks, Iain J. Gallagher, Rachel Crockett, Paul Dudchenko, Angus M. Hunter

**Affiliations:** 1 Department of Psychology, University of Stirling, Stirling FK9 4LA, UK; rachel.crockett@stir.ac.uk (R.C.); p.a.dudchenko@stir.ac.uk (P.D.); 2 MRC/Wits Developmental Pathways for Health Research Unit, Faculty of Health Sciences, University of the Witwatersrand, Johannesburg 2000, South Africa; ae.mendham@uct.ac.za (A.E.M.); julia.goedecke@mrc.ac.za (J.H.G.); lisa.micklesfield@wits.ac.za (L.K.M.); 3 Division of Exercise Science and Sports Medicine, Department of Human Biology, University of Cape Town, Cape Town 7700, South Africa; 4 Faculty of Health Science and Sport, University of Stirling, Stirling FK9 4LA, UK; tmzsim@gmail.com (S.A.T.); n.e.brooks@stir.ac.uk (N.E.B.); i.j.gallagher@stir.ac.uk (I.J.G.); a.m.hunter1@stir.ac.uk (A.M.H.)

**Keywords:** sarcopenia, osteoporosis, ageing, compositional analysis, moderate-to-vigorous physical activity, bone mineral density, grip strength, gait speed, accelerometry, muscle mass

## Abstract

This study aimed to investigate differences in physical activity (PA) patterns and the associations between objectively measured 24-h movement behaviors and musculoskeletal measures (muscle strength, muscle mass, physical performance, and bone mineral density) in a high-income and a low-income community. This cross-sectional study recruited independent living older adults aged 60–85 years from high-income Scottish (n = 150) and low-income South African (n = 138) settings. Participants completed demographic and health questionnaires, and testing included body composition and bone mineral density (dual energy X-ray absorptiometry), physical performance (grip strength, gait speed), and PA (accelerometry). Participants accumulated similar amounts of weekly total PA, however, the Scottish cohort engaged in more moderate-to-vigorous intensity PA (MVPA) and sedentary behavior (SB), while the South African cohort spent more time sleeping and in light intensity PA (LPA). From compositional data analysis, more time spent in MVPA relative to the other movement behaviors was positively associated with higher muscle mass (*p* < 0.001) and strength (*p* = 0.001) in the Scottish cohort. Conversely, more time spent in MVPA was associated with faster gait speed (*p* < 0.001) and greater hip bone mineral density (*p* = 0.011) in the South African cohort. Our findings confirm the beneficial role of MVPA in both high- and low-income cohorts, however, the relationship MVPA had with components of musculoskeletal health in older adults differed between settings.

## 1. Introduction

The number of older adults and years lived with disabilities is increasing worldwide [1]. Primary features of ageing include decreased bone and muscle health, which contribute to reduced quality of life and loss of mobility [2]. Decreased bone mass and changes in bone integrity are referred to as osteoporosis, a condition associated with greater fracture risk and reduced mobility [3]. Sarcopenia is a progressive and generalized skeletal muscle disorder that affects physical functionality and is identified by low muscle strength, along with decreased muscle mass [4]. As such, osteoporosis and sarcopenia are common disorders in older adults and prevention strategies should be a main focus to maintain quality of life with ageing.

In older adults, regular physical activity (PA) can increase functional independence, enhance immunity (a critical factor in the current COVID-19 pandemic), reduce obesity, and improve metabolic health and overall quality of life [5,6,7,8,9]. Specifically, allocating more time to higher PA intensities (moderate-to-vigorous PA, MVPA) by reducing time in sedentary behavior (SB) or light PA (LPA) has demonstrated a positive impact on musculoskeletal components and subsequent reductions in sarcopenia [10] and osteoporosis [11] rates in older adults. Assessing PA and SB is important as low levels of PA are recognized as underlying mechanisms of sarcopenia and osteoporosis [12,13]. Recent studies investigated the crosstalk between muscle and bone tissues due to their shared role in movement activities [14]. This interplay between muscle and bone supports the idea that the mechanical load produced by PA via muscle contraction shapes bone structure and maintains bone mineral density (BMD) and bone integrity with ageing [15]. This evidence highlights the need to consider bone, skeletal muscle, and their crosstalk in research with a view to inform physical activity guidelines in older adults.

The World Health Organization (WHO) recently introduced PA and SB guidelines for people of all ages [16]. The guidelines for older adults now consider movement behaviors throughout the day. Older adults’ PA patterns and intensities are often dictated by socioeconomic status, with differences shown between low- to middle-income countries (LMICs) and high-income countries (HICs) [17,18,19]. In HICs, there is easier access to facilities for leisure-time activities that include gym-based exercise and sports [20], which are activities associated with higher PA intensities, particularly MVPA [21]. Conversely, older adults in LMICs often accumulate PA through occupational and ambulatory activities typically completed at low intensities [22]. Notably, data from South African women indicate that in addition to high levels of LPA, there is also evidence of longer sleeping times (>9 h per night) associated with negative health outcomes [23]. Additionally, LMICs are often characterized by both a higher prevalence of obesity [24] and communicable and non-communicable diseases [25] compared to HICs [26]. Differences in living conditions and sociodemographic factors contribute to greater exposure and vulnerability of low socioeconomic communities to chronic diseases [27], leading to differences in general health status across socioeconomic settings. However, little is known on how differences in health status and movement behaviors in LMICs and HICs can affect the musculoskeletal health of men and women living in these settings.

In assessing movement behaviors, one faces methodological challenges that arise from the non-independence of these activity measures. Movement behaviors include all activities over a 24-h period across the movement spectrum, from no/little movement (sleep, SB) to movement of greater intensities (LPA and MVPA). Importantly, time spent in each movement behavior is related to the time spent in the other movement behaviors. Indeed, increasing the relative time spent in one behavior will result in a decrease in another as they are measured in a discrete period of time (24 h). However, the majority of statistical approaches used to analyze movement behaviors do not consider the 24-h period and the intrinsic collinearity of time use components. To address this in the current study, we used compositional data analysis (CoDa), which considers the co-dependence of movement behaviors [28].

Differences in movement behaviors between socioeconomic settings may suggest a different allocation of specific PA behaviors and represent an important target to improve research translation of behavior into context-specific population health. We had data for an LMIC (South Africa) and an HIC (Scotland). To date, no study has explicitly explored the relationship of all movement behaviors with muscle and bone integrity in older adults of similar age living in diverse settings in South Africa and Scotland. Further, there is limited research reporting objectively measured sleep in South African and Scottish older adults.

Accordingly, this study aimed to investigate differences in PA patterns and the associations between objectively measured 24-h movement behaviors and musculoskeletal outcomes (muscle strength, muscle mass, physical performance, and BMD), and their crosstalk, in representative samples of older adults from a high-income and a low-income country. On the basis of previous literature, we hypothesized that movement behaviors, particularly PA, would have a similar volume but would differ in intensity between older adults from high and low-income countries, with the high-income country displaying higher intensities of PA. Further, differences in health status and obesity prevalence may lead to different associations between physical activity patterns and musculoskeletal outcomes in high- and low-income communities.

## 2. Materials and Methods

In this cross-sectional study, a representative sample of Scottish older adults (n = 151) from a high-income urban setting and South African older adults (n = 154) from a low-income urban setting were recruited. These settings were categorized according to the World Bank classification [29]. Inclusion criteria were the same for the 2 cohorts: Participants were aged 60–85 years (older adult classification from the United Nations [30]), were ambulatory, living independently, and able to understand verbal and written information about the respective studies. Participants with diabetes were excluded from the Scottish study as this was required by the Scottish Ethics committee. The Scottish study was approved by the “West of Scotland Research Ethics Committee 3” (19/WS/0118). The South African study was approved by the Human Research Ethics Committee of the Faculty of Health Sciences at the University of Cape Town (HREC REF:095/2018), and the NHS, Invasive or Clinical Research Committee at the University of Stirling (NICR:17/18). All participants provided verbal and written informed consent prior to commencing the study. The study was conducted in accordance with the 1964 Declaration of Helsinki and later amendments [31].

### 2.1. Study Design

**Figure 1:**
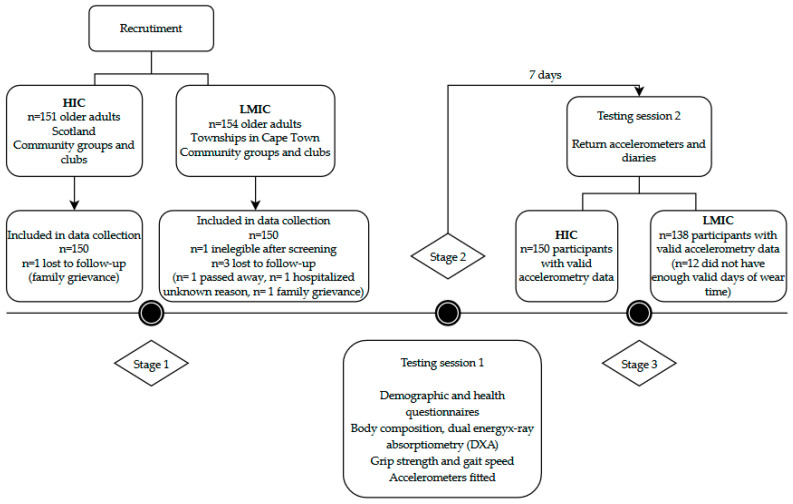
Recruitment and study design details.

Data collection protocols and methods for analysis were the same in the South African and Scottish studies unless otherwise stated. Participants from both samples were reimbursed for travel costs. Data were collected over 2 testing sessions (Figure 1). The first testing session was conducted over the course of 4 h, which included the following tests: demographic and health questionnaires. These included information on housing density (ratio of the number of people living in the house relative to the number of rooms) and smoking status (current and past smoking behaviors), as well as car ownership, civil status, employment, and sleep quality. Testing included body composition, BMD, hand grip strength, and gait speed, and objectively measured movement behaviors. Participants then attended a second testing session to return accelerometers after 7 days. In South Africa, data collection was undertaken by an exercise physiologist and 2 clinical research assistants. In Scotland, data collection was completed by a physiotherapist. In our final analysis (Figure 1), no participants were excluded in the Scottish sample (total n = 150), however, 12 older adults were excluded in the South African cohort due to the lack of valid accelerometry data (total n = 138).

### 2.2. Body Composition

Body mass was measured to the nearest 0.1 kg using a digital weighing scale (Scotland: Soehnle Connect, Soehnle-Waagen GmbH and Co.KG, Murrhardt, Germany; South Africa: BW-150, NAGATA, Tainan, Taiwan) in light-weight clothing without shoes, and height was assessed to the nearest 0.1 cm using a portable stadiometer (Scotland: Seca 213, Birmingham, UK; South Africa: 3PHTROD-WM, Detecto, MO, USA). Body mass index (BMI) was computed as body mass (kg)/height (m^2^) and classified according to World Health Organization (WHO) criteria [32].

Whole and subtotal (whole-body minus head) body composition, including fat-free soft tissue mass (FFSTM), fat mass, and BMD were measured using dual energy X-ray absorptiometry (Scotland: iDXA; GE Encore, version 13.40.038, GE Healthcare, Madison, WI, USA; South Africa: DXA; Discovery-W^®^, version 12.7.3.7, Hologic, Bedford, MA, USA) according to standard procedures. Appendicular skeletal muscle mass (ASM), the sum of FFSTM (kg) of both legs and arms, was adjusted for BMI (ASM_BMI_). BMD was quantified using DXA at the lumbar spine (lumbar vertebrae L1-L4), total hip, and femoral neck.

### 2.3. Sarcopenia and Bone Mineral Density Cut Points

To assess sarcopenia components, we considered grip strength adjusted for BMI (grip strength_BMI_) as our measure of muscle strength, and ASM adjusted for BMI (ASM_BMI_) as our measure of muscle mass, as used by the Foundation for the National Institutes of Health (FNIH) Sarcopenia Project [33]. Recommendations for cut points for weakness and low muscle mass included grip strength_BMI_ of <0.1 for men and <0.56 for women, and ASM_BMI_ of <0.789 for men and <0.512 for women, respectively [33]. Participants presenting with both weakness and low muscle mass were classified as sarcopenic. The recommended cut point for gait speed is ≤0.8 m∙s^−1^ [33].

The International Society for Clinical Densitometry (ISCD) guidelines for adults aged ≥50 years were used to determine those with osteopenia (t-score −2.5 to −1) and osteoporosis (t-score < −2.5) at 1 or more bone sites [34,35].

### 2.4. Hand Grip Strength and Functional Fitness Tests

Grip strength (kg) was measured with a hand dynamometer (Scotland: T.K.K.5001, Grip-A, Takei, Tokyo, Japan; South Africa: T.K.K. 5401, Grip-D, Takei, Tokyo, Japan). Measures were taken on the non-dominant side, while the participants were in a seated position with their elbow by the side at 90° angle position. The test was repeated 3 times with a 1-min rest between each effort. The maximum score achieved was used in the analysis [36].

Gait speed was assessed via a 6-m walk test. The participants were instructed to walk at a fast pace between two markers set 10 m apart. Time was measured manually for a 6 m distance (from 2 m to 8 m) to avoid influences of acceleration and deceleration. The mean score (m·s^−1^) from 3 measurements was recorded [37].

### 2.5. Movement Behaviors

Movement behaviors (MVPA, LPA, SB, sleep) were measured using ActiGraph GT3X+ (ActiGraph, Pensacola, FL, USA) accelerometer for 7 consecutive days. The device was worn over the right hip with an elastic belt [38] and initialized to collect data at 80 Hz. On the day of the trial, a trained researcher provided verbal, visual, and written instructions on how to wear the monitor correctly and checked participant self-attachment. Participants wore the monitor 24 h/day removing only for water-based activities (as bathing, swimming, or showering). Participants were asked to complete a written log to indicate times when the device was not worn. A second log was used to collect information on sleep behavior including time going to sleep and waking up. Activity counts were measured in 60-s epochs, and data were processed using ActiLife 6 software (ActiGraph Inc, Pensacola, FL, USA).

### 2.6. Accelerometry Data Management

Periods of nocturnal sleep were identified by visual inspection of ActiLife software outcomes [39]. Sleep diaries were used to check participant reported sleep times where nocturnal sleep periods were difficult to identify. Nocturnal time in bed (TiB, minutes) was used as the primary measure of sleep and it was used for analysis. To be included in the analysis, a participant needed to have at least 3 valid sleep nights (defined as >160 min of sleep time). Following the identification of nocturnal sleep periods, the remaining accelerometry wear time was checked for the other movement behaviors (PA and SB). A valid PA and SB dataset comprised of at least 4 days of valid wear time (with at least 1 weekend day). A single valid day was defined as a day with 600 min of wear time, following the exclusion of any other non-wear periods (non-wear time was defined as 60 min of consecutive zeros with allowance for 2 minutes of activity counts between 0 and 100). SB, LPA, and MVPA were classified using established cut-offs, as previously used in NHANES: sedentary < 100 cpm, total PA ≥ 100 cpm, 100 < LPA < 2020, MVPA ≥ 2020 cpm [40]. The duration of time spent in each activity behavior was determined (mins/day), and all behaviors accounted for whole participant daily time (1440 min).

### 2.7. Sleep and Physical Activity Recommendations

To assess sleep patterns, we classified participants according to National Sleep Foundation (NSF) recommendations [41]. Participants were considered as meeting sleeping recommendations if they accumulated 7–9 h of sleep per night, while older adults were considered to meet recommendations if their nocturnal sleep was between 7 and 8 h. Adults sleeping less than 6 h and older adults sleeping less than 5 h were considered as sleeping too little, while participants were sleeping too much if they accumulated more than 10 h of sleep for adults and more than 9 h of sleep for older adults.

Participants were classified as meeting PA guidelines according to the most recent WHO guidelines [16] on aerobic PA (do at least 150–300 min of moderate-intensity aerobic PA; or at least 75–150 min of vigorous-intensity aerobic PA; or an equivalent combination of MVPA, throughout the week).

### 2.8. Sleep Quality

The Pittsburgh Sleep Quality Index (PSQI) was used to evaluate sleep quality. The PSQI has been previously validated in high- [42] and low-income settings [43]. It consists of 19 self-rated items referring to the previous 4 weeks. This questionnaire provides a total score (>5 indicates poor sleep quality) along with seven sub-components: habitual sleep efficiency, subjective sleep quality, sleep disturbances, use of medication, daytime dysfunction, and sleep latency. Each of the 7 components have a range of 0–3 points where “0” indicates no difficulty and “3” indicates severe difficulty. Cronbach’s alpha internal consistency for this questionnaire in the Scottish sample was 0.75, while in the South African cohort was 0.71.

### 2.9. Statistical Methods

Analysis was conducted using IBM SPSS Statistics for Windows, version 25 (IBM Corp., Armonk, NY, USA), and compositional analyses were conducted using the open-source software Physical Activity CoDa Regression Model (PACRM) [44].

Traditional descriptive statistics were stratified according to Scottish and South African groups. The normality of the data was analyzed by the Kolmogorov–Smirnov test combined with Q-Q plots. Regression assumptions were checked, and in all models, non-normally distributed dependent variables were transformed with the most appropriate transformation. Descriptive statistics for continuous variables were expressed as mean and standard deviation (SD) or median and interquartile range (IQR) as appropriate. Categorical variables were expressed as frequency and percentage. PA variables were presented as mean and standard deviation (SD) or median and interquartile range (IQR) as appropriate in Table 1. For compositional data analysis, compositional means of movement behaviors were considered (Table S1). χ^2^ (chi-squared) or Fisher’s exact test (where appropriate) was conducted to determine differences in sleep sub-components of the PSQI (Table S2).

A compositional multivariate analysis of variance (MANOVA) on isometric transformed data was used to determine differences in time use composition between Scottish and South African participants (independent variable) [45]. To interpret differences between movement behaviors, we developed bootstrap percentile 95% confidence intervals [45]. Prior to MANOVA calculations, assumptions for this test were checked, and as they were not violated, we firstly calculated unadjusted MANOVA model and later considered covariates on the basis of previous literature [46]. Covariates in the model included: age, sex, BMI, education, and smoking status, as well as human immunodeficiency virus (HIV) and diabetes presence in the South African sample.

To investigate the associations between proportions of time spent in the movement behaviors and musculoskeletal outcomes, we then computed linear regression models separately for both cohorts. For each cohort, 4 compositional linear regression models were performed for each sarcopenia component (grip strength_BMI_, ASM_BMI_, and gait speed) and BMD site (femoral neck BMD, total hip BMD, and lumbar spine BMD) as response via isometric log-ratio (ilr) transformation of the time-use composition (explanatory variables) along with covariates. First, the composition of time spent in sleep, SB, LPA, and MVPA was considered. A set of 3 ilr-coordinates was obtained via sequential binary partition. Model *p*-values and *R^2^* coefficients were calculated from unadjusted linear regression models to assess the presence of a statistically significant relationship between time use composition and musculoskeletal components. The first coefficient (γ) and the corresponding *p*-value for each of the 4 linear regressions were used to determine if the time spent in a specific behavior relative to the others was significantly associated with grip strength_BMI_, ASM_BMI_, gait speed, and BMD. We further adjusted BMD models for sarcopenia components due to the crosstalk between these environments [14]. Outcomes from CoDa analysis should not be interpreted in isolation due to their nature (ratios between behaviors); regression coefficients should be considered relative to the remaining behaviors. Statistical significance was set at *p* < 0.05.

## 3. Results

### 3.1. Participant Characteristics

Age and sex did not differ between the South African and Scottish cohorts. However, compared to the Scottish cohort, South African participants reported a lower socioeconomic status, which was reflected in a higher housing density, lower level of education, and lower car ownership (Table 1). Smoking behavior and civil and employment status did not differ between the two cohorts. In the South African group, 22.5% of participants had diabetes and 8.7% were living with HIV.

**Figure 2:**
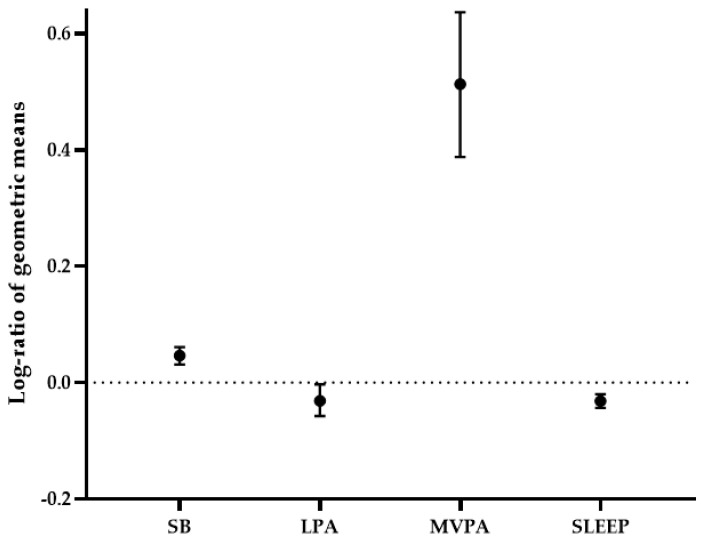
Log-ratio (95% confidence intervals) difference between the Scottish and South African groups for each movement behavior. Lines falling above the dotted line indicate that relative time spent in the specific behavior was higher in the Scottish cohort compared to South African cohort. Conversely, lines falling below the dotted line indicate that the component was higher in the South African sample.

Scottish and South African older adults accumulated similar amounts of total PA over the 7 days and showed significant differences between groups across all movement behaviors (Table 1 and Table S1) and 24-h time-use composition (Figure 2). The geometrics means, used for the 24-h time-use composition analysis, are shown in Table S1. In particular, Scottish participants spent more time in MVPA and SB compared to their South African counterparts. Alternatively, South African older adults spent more time in LPA and had longer nocturnal sleep duration. According to the most recent WHO recommendations for PA, the threshold of 150 min of combined MVPA was achieved by 51.4% of the Scottish cohort and 20.3% of the South African cohort. Among individuals meeting WHO PA recommendations, 18.7% of the Scottish and 6.5% of the South African older adults completed 300 min or more of MVPA during the week.

No significant differences were found in the number of people meeting NSF sleep recommendations in the two cohorts (Table 1). However, a higher percentage of Scottish older adults had an appropriate number of sleeping hours (50.7% vs. 25.5%, *p* < 0.001), while a greater percentage of South African participants were sleeping too long (45.4% vs. 22.0%, *p* < 0.001). Alongside longer nocturnal sleep, the South African cohort reported a greater number of sleep disturbances and daytime dysfunction, higher use of sleeping medication, and worse sleep latency, but greater sleep efficiency than the Scottish older adults (Table S2). However, no differences in the overall PSQI score were found between cohorts.

### 3.2. Body Composition, Bone Mineral Density, and Functional Measures

Body composition differed between the two cohorts (Table 2), with South African older adults having higher fat mass (kg and %) and BMI, and Scottish participants having higher FFSTM and ASM_BMI_. However, unadjusted ASM did not differ between cohorts. Significant differences were found in functional outcomes, with Scottish older adults displaying higher grip strength and grip strength_BMI_ and South Africans having a faster gait speed. According to the BMI adjusted FNIH cut-off, the South African group had a higher prevalence of sarcopenia than the Scottish cohort (30.4% vs. 2.0%, *p* < 0.001). Higher rates of osteopenia were found in the Scottish cohort, while higher rates of osteoporosis were found in the South African cohort (Table 2).

### 3.3. Relationship between 24-h Time Use Composition and Musculoskeletal Health in South African and Scottish Older Adults

Compositional linear regression models in relation to sarcopenia components and BMD are shown in Table 3. In the South African cohort, more time spent in MVPA relative to the other behaviors was associated with higher gait speed and total hip BMD. When adjusting for grip strength, ASM, and/or gait speed, we found that MVPA remained positively associated with total hip BMD in the South African cohort (Table 4). Therefore, the relationship between relative time spent in MVPA and total hip BMD was independent of sarcopenia components. When additionally adjusting the model for grip strength and ASM (Table 4), we found a positive association between relative time in MVPA and femoral neck BMD; however, when gait speed was included as a covariate, the positive association with femoral neck BMD was no longer significant.

In contrast, the Scottish cohort showed that higher relative time spent in MVPA was positively associated with higher grip strength_BMI_ and ASM_BMI_ (Table 3). No associations between specific movement behaviors and BMD were found. When adjusting the model for grip strength, ASM, and/or gait speed (Table 5), we did not find any association between 24-h time use composition and BMD outcomes. However, when adjusting for only grip strength, we found that the relative time spent in SB was negatively associated with femoral neck BMD.

In both cohorts, the overall 24-h time use composition accounted for a similar proportion of the variance for grip strength and ASM (Table 3). Conversely, in the South African cohort only, a significant association between movement behaviors and gait speed was reported. Time use composition explained almost 35% of the variance in gait speed in this cohort, with MVPA significantly dictating the association. The overall 24-h composition explained a similar amount of variance for femoral neck BMD in both cohorts (±7%). However, an association between overall 24-h composition and hip total BMD was only reported the South African cohort and accounted for ±10% of the variance.

## 4. Discussion

In the current study, we assessed and compared movement behaviors of older adults from low-income South African and high-income Scottish communities and examined associations with various measures of musculoskeletal health. Although the total amount of weekly PA between the two cohorts was similar, there were differences in the relative time spent in all behaviors, with Scottish participants spending more time in SB and MVPA and South African older adults spending more time in LPA and nocturnal sleep. We found a positive association between relative time in MVPA and sarcopenia components and BMD, but these differed between the cohorts. Specifically, higher MVPA time relative to the other behaviors was associated with higher ASM_BMI_ and grip strength_BMI_ in the Scottish cohort, and higher gait speed and total hip BMD in the South African cohort. Our results suggest that interventions targeted at older adults should consider the importance of engaging in higher intensities of PA patterns irrespective of the sociodemographic profile. However, the relationships between MVPA and components of musculoskeletal health were different between cohorts.

The first important finding was that the representative samples of older adults from the high-income and the low-income countries had significantly different time allocation in 24-h movement behaviors. Specifically, Scottish older adults displayed higher relative time in MVPA compared to the South African counterpart. This finding agrees with previous evidence highlighting high engagement in MVPA in high-income settings [17]. Conversely, we reported low time spent in MVPA relative to the other behaviors in the South African sample, a common issue of LMICs [21]. This finding could be explained by the fact that most of the participants involved in our study were either unemployed or retired. The main contributor to engagement in MVPA in LMICs is the occupational domain and to lesser extent participation in leisure activities [47,48]. In agreement with previous studies [49], South African older adults spent more time in LPA relative to the other movement behaviors. Thus, the understanding of population’s socioeconomic context is fundamental to translate evidence into appropriate policies and interventions.

Our finding of a beneficial association between MVPA and sarcopenia components confirms the importance of PA intensity on muscle mass, muscle strength, and physical performance. Our observations agree with recent studies involving older adults from Swedish and Tasmanian cohorts [46,50]. However, the novelty of our findings is in the consideration of the time spent across the entire 24-h day, while previous studies considered different movement behaviors in isolation. Regular weekly MVPA has been found to reduce sarcopenia risk and to be consistently associated with reduced incidence of these components [46]. Additionally, a previous study highlights a reduction in sarcopenia prevalence by displacing 15 min of either SB or LPA in favor of MVPA in older adults [10]. However, this study did not consider the effect of the whole 24-h spectrum on movement behaviors.

While in the Scottish cohort, MVPA was positively associated with both ASM_BMI_ and grip strength_BMI_, in the South African cohort, the relative time spent in MVPA was positively associated with gait speed. Although the muscular outcomes positively associated with MVPA are different between the cohorts, the findings overall suggest a protective role of higher PA intensities on functional outcomes. Differences in functional outcomes between the two cohorts could be due to differences in modalities and activities to achieve higher PA intensities. Scottish older adults engage in MVPA mostly for recreational and exercise purposes with gym-based exercise and sports (involving also upper body usage) [21], potentially resulting in greater values of grip strength and muscle mass. In contrast, the main contributors to PA in the South African cohort are active travel and occupational duties [22], leading to greater engagement of lower body functionality. Consequently, our results confirm the importance of MVPA for muscular health in older adults when considered relative to other movement behaviors. However, the influence of PA domains (leisure and occupational) among groups of older adults in different socio-economic settings should be considered.

The present study shows associations between movement behaviors and hip BMD in the South African cohort. Our results agree with previous studies reporting greater hip and femoral neck BMD with the engagement in habitual PA and MVPA [51]. The reason for the beneficial effect of PA on hip BMD could be related to greater cortical bone in femoral neck and total hip components, which is more susceptible to mechanical loading and absorption of ground reaction forces [52,53]. Alternatively, the density of bones at the lumbar spine, due to the predominance of trabecular bone, is more sensitive to the metabolic milieu rather than mechanical loading [54]. Total hip BMD was positively associated with relative time in MVPA in the South African cohort, but not in the Scottish cohort. Most of the PA performed in the South African group is low intensity walking for transport [48], leading to a greater influence and protective effect of PA on hip bones. Additionally, when adjusting our models for sarcopenia components in the South African cohort, the association between MVPA and total hip BMD remained significant, highlighting the independence of this relationship from muscular and functional factors. Despite this association, in our study, the prevalence of osteoporosis was still high in the South African cohort. This could be explained by the large proportion of South African older adults not meeting PA guidelines, which can lead to a decline in bone integrity; however, other risk factors for osteoporosis, such as nutrition, should be acknowledged. Thus, the importance of meeting PA recommendations should be reinforced in older adults from LMICs. However, the ideal amount of MVPA to prevent a decline in BMD is still poorly defined as the dose–response association between MVPA and BMD is not fully understood [55]. Indeed, multiple exercise types (involving balance, endurance, resistance, and functional exercise) appear to be more effective for maintaining bone health and reducing the risk of fracture [56]. Future longitudinal and intervention studies are required to address this research gap.

Previous data have shown that time spent sitting is negatively associated with hip BMD [57], and we have shown that the association between SB and femoral neck BMD was mediated by grip strength in Scottish older adults. Grip strength is well recognized as a proxy for physical activity [58] and is consistently associated with BMD across sites [59,60]. Additionally, grip strength was also a significant contributor to the model, further supporting the idea that the association between BMD and SB is mediated by muscle strength. This finding reinforces the idea of the crosstalk between muscle and bone, suggesting a shared pathological process in older adults [14,61]. Previous studies reported the negative impact of SB, independently of PA, on bone integrity [62]. Interestingly, when considering the whole 24 h spectrum in the Scottish cohort, SB was the only behavior associated with BMD and this was only observed after adjusting for grip strength. Conversely, higher relative daily time spent in MVPA was associated with better hip total bone health outcomes in the South African cohort. However, when muscle mass and muscle strength were considered in the model, femoral neck BMD was also positively associated with relative time spent in MVPA. This association further reinforces the presence of a muscle-bone unit, suggesting that future interventions should target engagement in resistance training [63] involving also upper body activities in older adults in both HIC and LMIC.

Our data suggest that MVPA was associated with better musculoskeletal health in both cohorts; however, this poses a challenge as a low percentage of South African older adults met PA guidelines as indicated by their lower engagement in higher PA intensities or MVPA. A previous study [64] showed that barriers to MVPA in a low-income South African setting included the perception of poor facilities and safety issues in the neighborhood, making it harder to engage in recreational MVPA. Inequalities in accessing opportunities to engage in leisure PA occur in most LMICs, with poor and overcrowded environments, lack of adequate facilities, and safety representing major barriers to PA [65]. Thus, when targeting engagement in PA in LMICs, we need to acknowledge the associated challenges, with the potential aim of removing these barriers. Conversely, a large number of Scottish older adults were already engaging in appropriate amount of PA. However, they also spent ≈10 h of the day in SB, and in line with the most recent recommendations by the WHO, this may have detrimental effects on increased risk of developing a chronic disease and mortality [66]. To sustain beneficial effects that PA has on different health outcomes with ageing, we should support older adults to maintain PA levels while reducing SB, particularly during the COVID-19 pandemic.

Along with differences in PA, we also found differences in the relative time spent sleeping. A recent review suggested that shorter and longer sleepers are at greater risk of developing muscle failure and lower values of BMD, when considering sleep in isolation [67,68]. Conversely, when the whole 24 h day was considered, no relationship between sleep duration and musculoskeletal health was found. Furthermore, in line with previous findings [23], the South African cohort in the present study had longer sleeping duration when compared to the Scottish cohort. Analysis of the PSQI subcomponents showed that the South African participants reported more sleep disturbances, longer sleep latency, higher use of medication, and more daytime dysfunction. These results could be related to the higher housing density in the South African group (three times more than the number of people per room in Scotland). Indeed, previous studies have shown that 42% of the people living in South African townships indicated that between 5 and 10 people were living in the housing unit allotted to them [69], and specifically 81.1% were sharing sleeping space or bedroom [70]. Poor sleep quality and age-related changes in sleep patterns have been shown to have adverse effects on health during ageing, particularly on mental and physical health [71]. Therefore, attention should be given to sleep quality and appropriate sleep duration when investigating ageing, and further work on this topic is warranted.

### Strengths and Limitations

This study has several strengths, including the use of objectively measured movement behavior outcomes in both high- and low-income communities with a shared protocol. With CoDa, we considered the whole 24-h time use composition, allowing us to investigate the combined effect of movement behaviors on musculoskeletal outcomes. However, different machines were used for multiple measures (whole body composition, bone measures, and grip strength), which meant that a direct comparison across these measures was not feasible. Regardless, this does not preclude the comparison of associations between movement behaviors and musculoskeletal outcomes. Due to ethics requirements, older adults with diabetes were not included in the Scottish cohort. Consequently, our findings cannot be generalized to all HIC older adult population. However, we performed a sensitivity analysis excluding participants with diabetes and HIV in the South African cohort. The results obtained for group differences in 24-h movement behaviors and associations with musculoskeletal outcomes did not change (data not shown). The study is limited by its cross-sectional design, precluding any claims on causal inference from our results. Thus, findings from this study can identify potential future targets that need to be addressed in future intervention or longitudinal studies. Additionally, the current study did not collect data on cognitive functioning or impairments. Future studies should include cognitive screening, especially if including participants at risk. The participants included in this study were living independently and therefore it is less likely that cognitive impairment would have an impact on the results presented. Lastly, we did not take into account the role of habitual strength training, as we focused on aerobic physical activity and future studies should also consider the potential mode specific training effects on musculoskeletal determinants.

## 5. Conclusions

We highlight the importance of the relative time spent in MVPA across the whole day on musculoskeletal health in older adults in both LMIC and HIC. Higher daily time allocations to MVPA relative to other behaviors were associated with better musculoskeletal and bone health. While the beneficial effect of MVPA in isolation on muscle and bone integrity is already known, recent evidence argued whether this positive impact remained when the whole day is considered. Our findings confirm the protective role of MVPA on muscle mass, muscle strength, physical functioning, and BMD. Specifically, in the high-income Scottish group, higher time spent in MVPA relative to the other behaviors was associated with greater muscle mass and muscle strength, while relative time spent in SB, mediated by muscle strength, was the main contributor to lower BMD. Thus, national plans and policymakers should support older adults in HIC in maintaining appropriate volume and intensity of PA and reducing SB with ageing. Conversely, in the low-income South African setting, higher relative time in MVPA had a positive effect on physical functioning and BMD. However, the presence of barriers to PA in LMICs should be acknowledged with a focus on PA security, ensuring equality in the access to sufficient, safe, and enjoyable spaces for PA. Thus, an integrated approach including the promotion of high-intensity PA and equality in PA access is needed.

Accordingly, future interventions should target the knowledge around the importance of engaging in higher PA intensities with ageing for musculoskeletal health acknowledging the challenges in different socio-economic settings.

## Figures and Tables

**Table 1:** Descriptive characteristics of Scottish and South African cohorts.

Variables	Scottish Cohort (n = 150)	South African Cohort (n = 138)	*p*-Value
Age (years)	69 (66–73)	68 (64–71)	0.085
Sex (n, %), Females	117 (78)	113 (82)	0.463
Education (n, %)			**<0.001**
Less than secondary	13 (9)	72 (52)	
Completed secondary			
Completed tertiary	27 (18)	65 (47)	
Housing density			
	110 (72)	1 (1)	
	0.3 (0.2–0.3)	1 (0.6–1.4)	**<0.001**
Smoking status (n, %)			0.283
Never smoked	94 (63)	100 (73)	
Previous smoker	53 (35)	22 (16)	
Current smoker	3 (2)	16 (12)	
Car owner (n, %)	141 (94)	17 (12)	**<0.001**
Civil status (n, %)			0.228
Single	30 (20)	55 (40)	
Married or living with a partner	105 (70)	25 (18)	
Widowed			
	15 (10)	58 (42)	
Employed, yes (n, %)	3 (2)	0	0.095
Accelerometer total wear time (min/day)	913 ± 46	878 ± 80	**<0.001**
Total PA (min/week)	324 ± 64	334 ± 96	0.112
SB (min/day)	593 (546–638)	539 (491–587)	**<0.001**
LPA (min/day)	287 ± 55	318 ± 92	**0.001**
MVPA (min/day)	27 (15–44)	11 (3–21)	**<0.001**
Nocturnal TiB (min/night)	513 ± 45	555 ± 75	**<0.001**
SB (% daily wear time)	41 (38–44)	37 (34–41)	**<0.001**
LPA (% daily wear time)	20 ± 4	22 ± 6	**0.001**
MVPA (% daily wear time)	2 (1–3)	1 (0.2–2)	**<0.001**
Nocturnal TiB (% daily wear time)	36 ± 3	39 ± 5	**<0.001**
MVPA (min/week)	153 (88–265)	64 (18–128)	**<0.001**
Meeting PA guidelines ≥150 min/week (n, %)	77 (51)	28 (20)	**<0.001**
Meeting sleep recommendations (n, %)	41 (27)	32 (23)	0.498
Sleeping within the appropriate range (n, %)	76 (51)	36 (26)	**<0.001**
Sleeping too short (n, %)	0	6 (4)	**0.011**
Sleeping too long (n, %)	33 (22)	64 (45)	**<0.001**

All normally distributed and skewed data are reported as mean ± SD and median (IQR, 25–75th percentile), respectively. Movement behaviors data are reported as traditional mean or median (min/day) and %. Abbreviations: HIV, human immunodeficiency virus. PA, physical activity. SB, sedentary behavior. LPA, light physical activity. MVPA, moderate-to-vigorous physical activity. TiB, time-in-bed. *p*-values represent a significant difference between groups. Parametric and non-parametric (Mann–Whitney *U*, Kruskal–Wallis) independent *t*-tests were conducted on normally distributed and skewed data, respectively. Chi-squared test was used to determine differences in frequency of each variable between Scottish and South African cohorts. Statistically significant results (*p* < 0.05) are highlighted in bold.

**Table 2:** Body composition, and functional and musculoskeletal variables of Scottish and South African cohorts.

Variables	Scottish Cohort (n = 150)	South African Cohort (n = 138)	*p* Value
Body composition			
Height (cm)	164.7 ± 9.2	157.3 ± 7.2	**<0.001**
Body mass (kg)	69.0 (60.8–77.3)	79.3 (66.8–93.2)	**<0.001**
BMI (kg/m^2^)	25.7 (23.5–28.3)	31.7 (27.8–38.3)	**<0.001**
Underweight (n, %)	2 (1.4)	1 (0.7)	
Normal (n, %)	57 (38.0)	21 (15.2)	
Overweight (n, %)	69 (46.0)	32 (23.2)	
Obese (n, %)	22 (14.6)	84 (60.9)	
Fat mass (kg)	25.4 ± 8.9	34.8 ± 14.1	**<0.001**
Fat mass (%)	37.5 (31.6–41.3)	47.2 (40.7–51.8)	**<0.001**
FFSTM (kg)	40.1 (36.3–45.2)	37.2 (33.4–42.3)	**<0.001**
ASM (kg)	17.6 (19.5–27.5)	17.3 (15–20.4)	0.066
ASM_BMI_ (kg/m^2^)	0.7 (0.6–0.8)	0.5 (0.5–0.6)	**<0.001**
Sarcopenia (n, %)	3 (2.0)	42 (30.4)	**<0.001**
Functional and sarcopenia measures			
Grip strength (kg)	23.0 (19.5–27.5)	20.1 (17.0–23.8)	**<0.001**
Grip strength (kg) men	34.7 (30.0–39.5)	23.8 (19.8–29.0)	**<0.001**
Grip strength (kg) women			
	21.7 (18.5–24.8)	19.6 (16.8–22.9)	**0.001**
Grip strength_BMI_ (kg/m^2^)	0.9 (0.7–1.2)	0.6 (0.5–0.8)	**<0.001**
Grip strength_BMI_ (kg/m^2^) men	1.3 (1.2–1.5)	1.0 (0.8–1.2)	**<0.001**
Grip strength_BMI_ (kg/m^2^) women			
	0.9 (0.7–1.0)	0.6 (0.5–0.7)	**<0.001**
Gait speed (m/s)	1.5 (1.4–1.7)	1.6 (1.4–1.7)	**0.041**
Bone mineral density			
Femoral neck BMD (g/cm^2^)	0.854 ± 0.149	0.758 ± 0.161	
Total hip BMD (g/cm^2^)	0.905 ± 0.150	0.896 ± 0.177	
Lumbar spine BMD (g/cm^2^)	1.112 ± 0.208	0.940 ± 0.191	
Femoral neck BMD_HEIGHT_ (g/cm^2^)	0.515	0.482	
Total hip BMD_HEIGHT_ (g/cm^2^)	0.547	0.569	
Lumbar spine BMD_HEIGHT_ (g/cm^2^)	0.672	0.598	
Femoral neck T score	−1.3 (−1.7–0.6)	−0.8 (−1.8–0.1)	
Total hip T score	−1.1 (−1.7–−0.4)	−0.3 (−1.3–0.7)	
Lumbar spine T score	−0.1 (−0.9–−0.7)	−1.1 (−2.2–0.1)	
Osteopenia (n, %)	105 (70.0)	54 (39.1)	
Osteoporosis (n, %)	16 (10.7)	28 (20.3)	

All normally distributed and skewed data are reported as mean ± SD and median (IQR—25–75th percentile), respectively. Abbreviations: BMI, body mass index. FFSTM, fat-free soft tissue mass. ASM, appendicular skeletal muscle mass. ASMBMI, appendicular skeletal muscle mass adjusted for body mass index. Grip strength_BMI_, grip strength adjusted for body mass index. BMD, bone mineral density. *p*-values represent a significant difference between groups. Parametric and non-parametric (Mann–Whitney *U*) independent *t*-tests were conducted on normally distributed and skewed data, respectively. Chi-squared test was used to determine differences in frequency of each variable between Scottish and South African cohorts. Statistically significant results (*p* < 0.05) are highlighted in bold.

**Table 3:** Compositional linear regression for the associations between movement behaviors and musculoskeletal outcomes.

Variables	γ Sleep	*p* Value	γ SB	*p*-Value	γ LPA	*p*-Value	γ MVPA	*p*-Value	Model *R^2^*	Model *p*-Value
South African cohort										
Grip strength_BMI_ (kg/m^2^)	0.114	0.310	−0.147	0.123	0.020	0.745	0.013	0.435	**0.110**	**0.001**
ASM_BMI_ (kg/m^2^)	0.036	0.571	−0.057	0.289	0.019	0.582	0.002	0.856	**0.200**	**<0.001**
Log gait speed (m/s)	−0.035	0.399	−0.010	0.768	0.019	0.380	**0.026**	**<0.001**	**0.349**	**<0.001**
Femoral neck BMD (g/cm^2^)	−0.064	0.415	0.023	0.725	0.021	0.612	0.020	0.087	**0.070**	**0.021**
Total hip BMD (g/cm^2^)	−0.020	0.807	−0.012	0.863	0.001	0.974	**0.031**	**0.011**	**0.099**	**0.003**
Lumbar spine BMD (g/cm^2^)	−0.014	0.894	−0.037	0.664	0.066	0.220	−0.015	0.296	0.008	0.787
Scottish cohort										
Grip strength_BMI_ (kg/m^2^)	0.065	0.688	−0.116	0.377	−0.045	0.644	**0.097**	**0.001**	**0.122**	**<0.001**
ASM_BMI_ (kg/m^2^)	−0.039	0.589	−0.022	0.706	0.012	0.789	**0.049**	**<0.001**	**0.119**	**<0.001**
Gait speed (m/s)	0.100	0.450	−0.090	0.378	−0.012	0.876	0.007	0.773	0.049	0.063
Log femoral neck BMD (g/cm^2^)	0.132	0.071	−0.116	0.051	−0.038	0.393	0.022	0.105	**0.067**	**0.018**
Log hip total BMD (g/cm^2^)	−0.019	0.698	−0.029	0.458	0.044	0.131	0.004	0.696	0.013	0.583
Log spine BMD (g/cm^2^)	−0.043	0.438	0.030	0.503	0.025	0.445	−0.013	0.208	0.013	0.591

Compositional regression coefficients (γ) for each movement behavior represent the association for time spent in each behavior relative to all other behaviors. Model *p*-value and *R*^2^ are based on the unadjusted model. Regression coefficients and corresponding *p*-values were adjusted for age, sex, education, and smoking status. Gait speed and bone mineral density (BMD) variables were further adjusted for body mass index (BMI). Regression coefficients and corresponding *p*-values for South African sample were additionally adjusted for diabetes and human immunodeficiency virus (HIV) prevalence. Statistically significant associations (*p* < 0.05) are highlighted in bold.

**Table 4:** Compositional linear regression model for South African cohort between bone mineral density variables and movement behaviors considering bone-muscle crosstalk.

Variables	γ Sleep	*p* Value	γ SB	*p*-Value	γ LPA	*p*-Value	γ MVPA	*p*-Value
Model 1: additionally adjusted for grip strength								
Femoral neck BMD (g/cm^2^)	−0.080	0.257	0.019	0.748	0.039	0.326	**0.022**	**0.043**
Hip total BMD (g/cm^2^)	−0.048	0.512	0.002	0.984	0.015	0.724	**0.032**	**0.005**
Spine BMD (g/cm^2^)	−0.025	0.778	−0.029	0.707	0.064	0.205	−0.010	0.442
Model 2: additionally adjusted for ASM								
Femoral neck BMD (g/cm^2^)	−0.087	0.236	0.014	0.814	0.049	0.230	**0.024**	**0.029**
Hip total BMD (g/cm^2^)	−0.049	0.525	−0.002	0.980	0.015	0.720	**0.035**	**0.002**
Spine BMD (g/cm^2^)	−0.036	0.709	−0.038	0.629	0.081	0.125	−0.008	0.565
Model 3: additionally adjusted for gait speed								
Femoral neck BMD (g/cm^2^)	−0.078	0.277	0.014	0.815	0.042	0.292	0.021	0.064
Hip total BMD (g/cm^2^)	−0.039	0.596	−0.005	0.933	0.005	0.707	**0.029**	**0.017**
Spine BMD (g/cm^2^)	−0.022	0.815	−0.038	0.627	0.070	0.176	−0.011	0.464
Model 4: additionally adjusted for grip strength + ASM + gait speed								
Femoral neck BMD (g/cm^2^)	−0.086	0.244	0.021	0.725	0.043	0.301	0.022	0.056
Hip total BMD (g/cm^2^)	−0.041	0.590	0.007	0.913	0.004	0.922	**0.030**	**0.013**
Spine BMD (g/cm^2^)	−0.039	0.497	−0.026	0.740	0.070	0.190	−0.011	0.452

Compositional regression coefficients (γ) for each movement behavior represent the association for time spent in each behavior relative to all other behaviors. Regression coefficients and corresponding *p*-values were adjusted for age, gender, education, smoking status, body mass index (BMI), diabetes, and human immunodeficiency virus (HIV) prevalence. Models 1, 2, and 3 were respectively additionally adjusted for grip strength, appendicular skeletal muscle mass, and gait speed. Model 4 was additionally adjusted for all sarcopenia components. Statistically significant associations (*p* < 0.05) are highlighted in bold.

**Table 5:** Compositional linear regression model for Scottish cohort between bone mineral density variables and movement behaviors considering bone-muscle crosstalk.

Variables	γ Sleep	*p*-Value	γ SB	*p*-Value	γ LPA	*p*-Value	γ MVPA	*p*-Value
Model 1: additionally adjusted for grip strength								
Log femoral neck BMD (g/cm^2^)	0.113	0.056	−0.113	0.044	−0.045	0.287	0.025	0.067
Log hip total BMD (g/cm^2^)	−0.030	0.519	−0.019	0.602	0.042	0.137	0.007	0.430
Log spine BMD (g/cm^2^)	−0.038	0.485	0.033	0.449	0.017	0.603	−0.012	0.237
Model 2: additionally adjusted for ASM								
Log femoral neck BMD (g/cm^2^)	0.133	0.056	−0.108	0.053	−0.046	0.275	0.022	0.108
Log hip total BMD (g/cm^2^)	−0.030	0.512	−0.017	0.656	0.042	0.141	0.005	0.573
Log spine BMD (g/cm^2^)	−0.038	0.486	0.033	0.454	0.017	0.603	−0.012	0.250
Model 3: additionally adjusted for gait speed								
Log femoral neck BMD(g/cm^2^)	0.130	0.061	−0.109	0.051	−0.044	0.295	0.023	0.085
Log hip total BMD (g/cm^2^)	−0.031	0.509	−0.018	0.620	0.043	0.131	0.006	0.481
Log spine BMD (g/cm^2^)	−0.007	0.492	0.033	0.461	0.017	0.606	−0.002	0.247
Model 4: additionally adjusted for grip strength + ASM + gait speed								
Log femoral neck BMD (g/cm^2^)	0.134	0.054	−0.008	0.053	−0.009	0.252	0.023	0.094
Log hip total BMD (g/cm^2^)	−0.028	0.546	−0.018	0.635	0.040	0.162	0.006	0.519
Log spine BMD (g/cm^2^)	−0.376	0.490	0.032	0.461	0.018	0.601	−0.012	0.251

Compositional regression coefficients (γ) for each movement behavior represent the association for time spent in each behavior relative to all other behaviors. Regression coefficients and corresponding *p*-values were adjusted for age, gender, education, smoking status, and body mass index (BMI). Models 1, 2, and 3 were respectively additionally adjusted for grip strength, appendicular skeletal muscle mass, and gait speed. Model 4 was additionally adjusted for all sarcopenia components. Statistically significant associations (*p* < 0.05) are highlighted in bold.

## Data Availability

The datasets generated and/or analyzed during the current study are not publicly available due to data sharing guidelines from the funders. All data and supporting documentation are available from the corresponding author on reasonable request.

## Supplementary Materials

The following are available online at https://www.mdpi.com/article/10.3390/ijerph18084310/s1, Table S1: Movement behaviors MANOVA between Scottish and South African cohorts. Table S2: Pittsburgh Sleep Quality Index sub-components results for Scottish and South African cohorts.
