# Supplementary material for: Intensity Matters for Musculoskeletal Health: A Cross-Sectional Study on Movement Behaviors of Older Adults from High-Income Scottish and Low-Income South African Communities
Source: Int J Environ Res Public Health. 2021 Apr 19;18(8):4310. doi: 10.3390/ijerph18084310 (PMC8072994; doi:10.3390/ijerph18084310)
Supplement: Supplementary file 1 [file ijerph-18-04310-s001.zip › ijerph-1195322-supplementary.pdf]

International Journal of
Environmental Research and Public Health

MDPI

SUPPLEMENTARY MATERIAL

Table S1. Movement behaviours MANOVA between Scottish and South African cohorts.

Variables	Scottish	South African	P value
SB (min/day)	604	551	**0.020**
LPA (min/day)	288	312	**<0.001**
MVPA (min/day)	20	6	**<0.001**
Nocturnal TiB (min/day)	523	569	**<0.001**

Time use composition data are reported as compositional means (mins/day). Movement behaviours have been normalised to 1440 minutes. Abbreviations: SB, sedentary behaviour. LPA, light physical activity. MVPA, moderate-to-vigorous physical activity. TiB, time-in-bed. Results of Multivariate Analysis of Variance (MANOVA) of differences in time spent in sedentary behaviours, light physical activity, moderate-to-vigorous physical activity, and sleep between Scottish and South African groups, analysed using CoDa approach.

Table S2. Pittsburgh Sleep Quality Index sub-components results for Scottish and South African cohorts.

Variables		Scottish cohort (n=150)	South African cohort (n=138)	P value
Sleep efficiency (n, %)	0, no difficulty	52 (34.7)	73 (52.9)	**0.002**
	1, fairly good	34 (22.7)	25 (18.1)	
	2, fairly bad	40 (26.7)	16 (11.6)	
	3, severe difficulty	24 (16)	24 (17.4)	
Subjective sleep quality (n, %)	0, no difficulty	30 (20)	30 (21.7)	0.301
	1, fairly good	77 (51.3)	72 (52.2)	
	2, fairly bad	37 (24.7)	23 (16.7)	
	3, severe difficulty	6 (4)	10 (7.2)	
Sleep disturbances (n, %)	0, no difficulty	3 (2)	0	**<0.001**
	1, fairly good	114 (76)	48 (34.8)	
	2, fairly bad	31 (20.7)	73 (52.9)	
	3, severe difficulty	2 (1.3)	17 (12.3)	
Use of medication (n, %)	0, no difficulty	63 (42)	89 (64.5)	**<0.001**
	1, fairly good	82 (54.7)	2 (1.4)	
	2, fairly bad	5 (3.3)	18 (13)	
	3, severe difficulty	0	29 (21)	
Daytime dysfunction (n, %)	0, no difficulty	63 (42)	22 (15.9)	**<0.001**
	1, fairly good	82 (54.7)	53 (38.4)	
	2, fairly bad	5 (3.3)	53 (38.4)	
	3, severe difficulty	0	10 (7.2)	
Sleep latency (n, %)	0, no difficulty	37 (24.7)	33 (23.9)	**<0.001**
	1, fairly good	59 (39.3)	25 (18.1)	
	2, fairly bad	35 (23.3)	39 (28.3)	
	3, severe difficulty	19 (12.7)	41 (29.7)	

Results for sleep sub-components are reported as total number and percentage. Kruskal–Wallis test was used to determine differences in frequency of each variable between Scottish and South African cohort.
